# Bibliographical Notices

**Published:** 1854-11

**Authors:** 


*Elkoplasty, or Anaplasty applied to the Treatment of Old Ulcers; also, a New Mode of Treatment for Delayed or Non-Union of a Fractured Humerus.* By Frank H. Hamilton, A.M., M.D., Professor of Surgery, etc. New York, Holman, Gray & Co. 1854.

Such is the title of a pamphlet dedicated "To my Father and my Mother."

The author seriously proposes to cut out old ulcers, "although the health of the body and of the limb may be perfect," and to transplant a piece of skin from another portion of the body, or from another person, to the bed of the ulcer.

There may, perhaps, be some surgeons at the present day who believe that there is something mysterious in these affections, and who would receive with great doubt the statement, that *all ulcers can be healed*, provided there be no constitutional taint or direct local cause. By them the announcement of a process for their cure will be hailed with delight.

We are certain, however, that their confidence in this new remedy will be diminished when they become acquainted with the author's pathological views.

On the first page he announces that ulcers resulting from lacerated wounds, in which the skin is much torn, *will not heal even in healthy persons.* "In such cases the refusal of the ulcers to heal is entirely owing to the extensive loss of the integument."

The mere statement of such propositions will bring to the minds of those even, whose experience is not the most extensive, numerous cases of severe injuries by lacerated wounds, and by burns also, where the ulcer healed without difficulty, provided there was no constitutional vice, or local cause for its continuance.

Notwithstanding the erroneous views contained in this paper, it has been extensively copied by the press, and we are sorry to say, that its publication has not as yet met with a single rebuke.

Bad practice must always follow upon bad principles, and where the true basis of surgery is wanting, even professors become mere operators. We cannot lay down in this notice even the outline of the different processes upon which repair of tissues depend. We should have to explain the laws of healthy nutrition, and those of the reproduction of lost parts. It would require a discussion of the materials necessary for reparation, an appeal to the microscopic characters and changes, and, in fact, a short lecture on the cell doctrine and histology, for all of which we refer Prof. Hamilton to Mr. Paget, in his Lectures on Surgical Pathology.

We cannot refrain from giving another quotation from the author, which is quite characteristic, and upon which any comment is unnecessary:—" By this means I hope, gentlemen, not only to supply an amount of skin equal to the size of the piece transferred, but to furnish also a nucleus by which additional skin shall be formed. I hope to establish a new centre of life—an oasis, from whose outer verge a true and healthy vegetation shall advance in every direction over the exhausted soil." This is certainly poetical if not philosophical.

Should the reader require any further evidence of the author's original views we would refer him to p. 6, where he certainly will receive a novel idea upon the formation of epithelial cells. He says, "the *new vessels*, becoming more and more attenuated as they stretch inward from the periphery, lose, at length, the power of *generating epithelial cells*," &c.

After this sample of novelty, to say the least, we suppose that our readers will be sufficiently satisfied with Prof. Hamilton and his new modes of treatment, to excuse us from giving any analysis of his statements and theories upon ununited fractures of the humerus.

---

*Principles of Comparative Physiology.* By WILLIAM B. CARPENTER, M.D., F.R.S., F.G.S., Examiner in Physiology and Comparative Anatomy in the University of London, &c. *With three hundred and nine wood engravings. A new American, from the fourth and revised London Edition.* Philadelphia: Blanchard & Lea. 1854.

Many and numerous qualifications of mind are needed to make a good writer on physiology. He should, in the first place, be thoroughly acquainted with the existing state of his particular department; he should have the power of elaborating from the rich, though often crude and undigested stores of scientific information surrounding him, what is most needful for his own purposes; he should inculcate definite *principles*, illustrating them by the introduction of established facts alone; avoiding the tendency to hypothesis, so common to all writers on sciences not yet established, he should, still, be both original and suggestive; where any doubt exists upon a subject, he should, if possible, put it to the test of positive experiment, or else make such an exposition of the different views held in regard to it, as to enable his readers to understand both sides of the question; he should not allow himself to be betrayed into personalities or controversies, and should be ready to confess his errors, should more extended researches have proved his views to be incorrect; and to crown the whole, his diction should be worthy of his subject, clear, flowing, and to the point.

Such a combination of qualifications being so rare, our readers may be tempted to exclaim somewhat as did the Prince of Abyssinia on hearing Imlac's elaborate and comprehensive description of his own vocation, "Enough! thou hast convinced me that no human being can ever be a poet;" yet, we feel sure, they will agree with us that Prof. Carpenter has many, if not all of the above mentioned requisites. It is owing, in fact, to the possession of them, that he has done more to render the study of physiology popular with the profession than all of his cotemporaries united.

The present work, which in the previous edition was part of "The Principles of Physiology, General and Comparative," is for various reasons stated in the preface, published by itself. "It has been largely augmented and carefully revised throughout;" and the author thinks that it "more completely represents the state of the science at the period of its publication than it has done on any preceding occasion." To bring it into accordance with the results of recent enquiries, some of the chapters had either to be re-written or greatly modified. The article on nutrition and that on generation and development have undergone the greatest alterations. We shall only draw attention to one of these, the author's change of opinion respecting the signification of fibrin in the animal economy. In the preceding edition it was considered as the material "most completely prepared for organization, and which supplies what is requisite for the nutrition of the larger proportion of the solid tissues of the body;" the vital properties of the blood were thought, also, to be more dependent on it than upon any of the other constituents. In the present work much less importance is attached to its value, it being regarded as the special pabulum only of the gelatinous tissues, whose vital endowments are exceedingly low. The proportional amount of the components of the blood is also differently given in the present edition, the average proportion of blood-corpuscles being much greater and the albumen much less than formerly stated.

We regret that we have reached the limits allowed us for the present notice, and must, therefore, close by referring our readers to the work itself. We are confident that their perusal of it will be attended with both pleasure and profit.

The publishers deserve praise for the admirable style of its typography and paper. The wood cuts are excellent, being nearly, if not quite, equal to the English.

---

*The Principal Forms of the Skeleton and of the Teeth.* By PROFESSOR R. OWEN, F. R. S., &c. Philadelphia: Blanchard and Lea. 1854.

To the philosophical anatomist it is impossible to over-estimate the value of a correct understanding of the neuro-skeleton. It is in the construction of this system, as Prof. Owen well observes, that the most interesting and beautiful evidences of unity of plan, as well as of adaptation to end, have been observed. Dissimilar as are the structures of the different species and classes of the vertebrate kingdom, their relations to each other become at once comprehensible when we regard them as mere modifications of a common ideal or archetype. It is by this clue alone, too, that we can properly understand the osseous structure of man, its organization being otherwise unintelligible without the light thrown upon it by this splendid generalization.

The chief object of the present essay on "the Principal Forms of the Skeleton" is the inculcation of certain general anatomical principles, by which the student may be guided and facilitated in this department of science. Under the head of principles of osteology the author succinctly describes the composition of bones, their primary classification, the dermo-skeleton, growth of bones, structure of bones in different classes, the neuro-skeleton, the vertebræ and the archetype of the skeleton. The osteology of the fish and the adaptation of its skull and skeleton to aquatic life; the principal forms of the skeletons of reptiles; the skeleton of birds; different forms of the skeleton in the class mammalia; the necessity of general and special terms in osteology, and the progressive expansion of the cranium in mammalia are then considered as minutely as the limits of a popular treatise allow, each subject and different form being elucidated and made plain by outline illustration. The result of these extensive comparisons is the recognition of a governing archetype, the departures from which may be intelligibly explained. Great stress is laid upon the necessity of a correct nomenclature.

The following are the author's "Concluding Remarks:"

A retrospect of the varied forms and proportions of the skeletons of animals, whether modified for aquatic, aerial, or terrestrial life, will show that whilst they were perfectly and beautifully adapted to the sphere of life and exigences of the species, they adhered with remarkable constancy to that general pattern or archetype which was first manifested on this planet, as Geology teaches, in the class of fishes, and which has not been departed from even in the extremely modified skeleton of the last and highest form which Creative Wisdom has been pleased to place upon this earth.

It is no mere transcendental dream, but true knowledge and legitimate fruit of inductive research, that clear insight into the essential nature of each element of the bony framework, which is acquired by tracing them step by step, as *e. g.* from the unbranched pectoral ray of the lepidosiren to the equally small and slender but bifid pectoral ray of the amphiume, thence to the similar but trifid ray of the proteus, and through the progressively superadded structures and perfection of the limbs in higher reptiles and in mammals. If the special homology of each part of the diverging appendage and its supporting arch are recognizable from man to the fish, we cannot close the mind's eye to the evidences of that higher law of archetypal conformity on which the very power of tracing the lower and more special correspondences depend.

Buffon has well remarked, in the Introduction to his great work on Natural History: "It is only by comparing that we can judge, and our knowledge turns entirely on the relations that things bear to those which resemble them and to those which differ from them; so, if there were no animals, the nature of man would be far more incomprehensible than it is."

And if this be true, as to man's general nature and powers, it is equally so with regard to his anatomical structure.

In the same spirit our philosophic poet felt that—

> "'Tis the sublime of man,
> Our noontide majesty, to know ourselves
> Part and proportions of a wondrous whole."—COLERIDGE.

Vertebrated animals of progressively higher grades of structure have existed at successive periods of time on this planet, and they were constructed on a common plan with those that still exist.

Some have concluded, therefore, that the characters of a species became modified in successive generations, and that it was transmuted into a higher species; a reptile, *e. g.* into a mammal; an ape, into a negro. Let us consider, therefore, the import and value of the osteological differences between the gorilla—the highest of all apes—and man, in reference to this "transmutation hypothesis." The skeleton of an animal may be modified to a certain extent by the action of the muscles. By the development of the processes, ridges, and crests, the anatomist judges of the muscular power of the individual to whom a skeleton under comparison has appertained. A very striking difference from the form of the human cranium results from the development of certain crests and ridges for the attachment of muscles, in the great apes; but none of the more important differences, on which the naturalist relies for the determination of the genus and species of the orangs and chimpanzees, have such an origin or dependent relation. The great superorbital ridge, *e. g.* against which the facial line rests in Fig. 50, is not the consequence of muscular action or development: it is characteristic of the genus *Troglodytes* from the time of birth; and we have no grounds for believing it to be a character to be gained or lost through the operations of external causes, inducing particular habits through successive generations of a species.

No known cause of change productive of varieties of mammalian species could operate in altering the size, shape, or connections of the prominent premaxillary bones, which so remarkably distinguish the great *Troglodytes gorilla* from the lowest races of mankind. There is not, in fact, any other character than that founded upon the development of bone for the attachment of muscles, which is known to be subject to change through the operation and influence of external causes. Nine-tenths of the differences which have been cited (see the *Transactions of the Zoological Society*, vol. iii. p. 413), as distinguishing the great chimpanzee from the human species, must stand in contravention of the hypothesis of transmutation and progressive development, until the acceptors of that hypothesis are enabled to adduce the facts demonstrative of the conditions of the modifiability of such characters. Moreover, as the generic forms of the ape tribe approach the human type, they are represented by fewer species. The unity of the human species is demonstrated by the constancy of those osteological and dental peculiarities which are seen to be most characteristic of the *bimana* in contradistinction from the *quadrumana*.

Man is the sole species of his genus (*homo*)—the sole representative of his order (*bimana*); he has no nearer physical relations with the brute kind than those that belong to the characters which link together the unguiculate division of the mammalian class.

Of the nature of the creative acts by which the successive races of animals were called into being, we are ignorant. But this we know, that as the evidence of unity of plan testifies to the oneness of the Creator, so the modifications of the plan for different modes of existence illustrate the beneficence of the Designer. Those structures, moreover, which are at present incomprehensible as adaptations to a special end, are made comprehensible on a higher principle, and a final purpose is gained in relation to human intelligence; for in the instances where the analogy of humanly invented machines fails to explain the structure of a divinely created organ, such organ does not exist in vain if its truer comprehension, in relation to the Divine idea, or prime Exemplar, lead rational beings to a better conception of their own origin and Creator.

We earnestly recommend those of our readers who desire further information on these matters to purchase this little volume. It embodies a very clear and concise account of the views and researches of the most distinguished osteologist of the present age, and will well repay their perusal.

